# Theranostic Advances in Breast Cancer in Nuclear Medicine

**DOI:** 10.3390/ijms22094597

**Published:** 2021-04-27

**Authors:** Nasim Vahidfar, Ayuob Aghanejad, Hojjat Ahmadzadehfar, Saeed Farzanehfar, Elisabeth Eppard

**Affiliations:** 1 Department of Nuclear Medicine, Vali-Asr Hospital, Tehran University of Medical Sciences, Tehran 1419733141, Iran; saeed.farzanefar@gmail.com; 2 Research Center for Pharmaceutical Nanotechnology, Biomedicine Institute, Tabriz University of Medical Sciences, Tabriz 51368, Iran; aghanejaday@tbzmed.ac.ir; 3 Department of Nuclear Medicine, Klinikum Westfalen, 44149 Dortmund, Germany; Hojjt.Ahmadzadefar@ruhr-uni-bochum.de; 4 Positronpharma SA. Rancagua 878, Santiago 7500621, Chile; elisabeth.eppard@med.ovgu.de; 5 Department of Nuclear Medicine, University Hospital Magdeburg, Leipziger Strass 44, 39120 Magdedurg, Germany

**Keywords:** theranostic, radiopharmaceutical, imaging, therapy

## Abstract

The implication of ‘theranostic’ refers to targeting an identical receptor for diagnostic and therapeutic purposes, by the same radioligand, simultaneously or separately. In regard to extensive efforts, many considerable theranostic tracers have been developed in recent years. Emerging evidence strongly demonstrates the tendency of nuclear medicine towards therapies based on a diagnosis. This review is focused on the examples of targeted radiopharmaceuticals for the imaging and therapy of breast cancer.

## 1. Introduction

Breast cancer (BC) is the most frequent malignancy among women worldwide [1,2,3]. It is demonstrated that nearly about 70–80% of primary BC can be cured. Treatments of advanced species with distant metastasis by using prevalent procedures are almost impossible [4]. Effective and impressive role of nuclear medicine in direct detection of BC initiated first in the early 1990’s when technetium-99m-methoxyisobutylisonitrile ([^99m^Tc]Tc-MIBI) was used for diagnosis of lesions of dense breasts which were not detectable by mammography [5]. Imaging facilities in nuclear medicine have made the diagnosis, staging, response and follow-up process evaluations convenient in a patient with BC [6]. Lately profitable progresses in therapeutic purposes of BC have been achieved. Based on molecular classification of BC, which can be categorised as luminal (A/B), HER2, basal like and breast like subtypes and also their prognosis, selective and specific radiopharmaceuticals can be designed and formulated [7].

**Figure 1:**
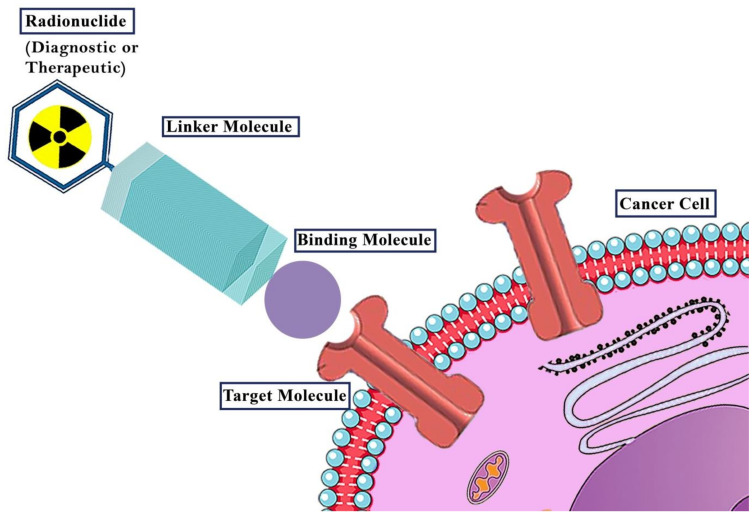
The general concept of theranostic radiopharmaceuticals. A radionuclide is combined with a targeting vector (Binding molecule). The choice of radionuclide defines the purpose of the radiopharmaceutical. γ^−^ or β^+^-emitters are used for diagnostics while β^−^- and α-emitters are applied in therapy. The targeting vector guides the radiopharmaceutical to its specific target (e.g., receptors). To combine the radionuclide with a target vector without reducing its affinity to its target, normally, depending on the type of radionuclide (e.g., metal, non-metal), linking structures (linker molecules) are necessary.

In metastatic cases, severe pain can be palliated with vast series of radiopharmaceuticals including strontium-89 (^89^Sr), samarium-153 (^153^Sm), phosphorus-32 (^32^P) and rhenium-186/188 (^186/188^Re) [5,8]. A relatively recent huge progress in nuclear medicine is the application of imaging agents for the evaluation of uptake and localisation, biodistribution, the related dose of therapeutic tracer and response to treatment. This remarkable concept is named ‘theranostic’ (Figure 1) [9]. This unique application (therapy + diagnostics) means combination of radiolabelled ligands with gamma-(γ) or positron-(β^+^) emitting radionuclides for diagnostic purposes with the same ligand incorporating therapeutic radionuclides such as alpha-(α) or beta-(β^−^) emitters to perform a personalised targeted treatment based on the preliminary diagnostic procedure [10,11,12]. Several successful theranostic approaches based on peptidomimetics, antibodies and small molecules have presented significant responses in clinical trials. Based on the accumulation mechanism of these radiopharmaceuticals, they can be useful for specific or extensive types of tumours [13].

The aim of this review article is to discuss the concept of theranostic radiopharmaceuticals with application in BC.

## 2. Recent Progresses for Diagnosis and Treatment of BC

BC is one of the major problems leading to death in women worldwide [14]. Each year American Cancer Society estimates new cases and deaths in United States. Evidence demonstrate that the greatest number of deaths are related to lung, prostate and colorectal cancers in men and lung, breast and colorectal cancers in women [14]. It is strongly believed that distant metastases are responsible for more than 90% deaths caused by BC [15]. However very impressive progressions in diagnosis and treatment of BC, have been done recently, but metastatic recurrences would be inevitable in 20–30% of patients [16,17]. Depending on the pathological source of the tumours, liver, lung, bone and brain should be considered as metastatic tissues [18,19]. Against definite opinions of many reports based on that, distant metastases known as secondary or late symptoms of BC, there are evidences that prove that distant metastases could be also an early symptom in some cases [20]. Early diagnosis may include some contemporary methods such as digital mammography (DM), magnetic resonance imaging (MRI) and molecular breast imaging (MBI) [21]. Through mammogram studies, in addition to x-ray exposure to the patients, numerous false positive results in many cases will require further evaluations via imaging or pathological assays [22,23]. Due to the poor selectivity against high sensitivity of MRI, and also its dependency to contrast agents, this method does not use as a routine diagnostic procedure. However according to the considerations of national comprehensive cancer network (NCC), MRI can be authorised in some specific cases [22,24]. MBI uses radioactive tracers for diagnosis of BC [21]. In mid 2000 s [^99m^Tc]Tc-sestamibi was used as MBI agent with gamma cameras as a reliable and selective method for functional imaging [25]. Nuclear medicine by providing of physiological patterns, plays a fundamental role in prognostic, staging and therapy of BC [26]. Molecular imaging with single photon emission computed tomography (SPECT/CT) and positron emission tomography/computed tomography (PET/CT) incorporation with radiolabelled molecules can be beneficial for staging, response evaluation, restaging, detection of recurrence and follow-up during or after cytostatic therapy for cancer management [27]. Besides that, therapeutic radiopharmaceuticals in nuclear medicine, compared to other conventional methods such as surgery, radiation therapy (RT), chemotherapy (CT) and endocrine (hormone) therapy (ET) are non-invasive and includes fewer side effects [21,28,29].

## 3. Theranostic Approaches for Cancer Management in Nuclear Medicine

The concept of radiotheranostics refers to 1941, when Saul Hertz at Massachusetts General Hospital (Boston, Mass) used radioiodine for thyrotoxicosis treatment [30]. This was the turning point in nuclear medicine and shortly thereafter, in 1942, the first publication of treatment of similar patients was published [31,32]. In order to evaluate effectiveness of the therapeutic procedure, the first diagnostic imaging with radioiodine was performed in 1950 at the University of California, Los Angeles (UCLA) [33]. Pre-targeting verification includes sensitivity, specificity and quantification imaging studies to prove the therapeutic feasibility of a specific radiotracer [34].

Nowadays, this exclusive integration of diagnosis and therapy are common. The most recent progress in this precedent is the administration of those radiotheranostic agents which target somatostatin receptors (SSTRs) in neuroendocrine tumours (NETs), human epidermal growth factor receptor 2 (HER2) antigens in BC and the prostate specific membrane antigen (PSMA) in prostate cancer (PC) [30,34,35]. The two prevalent ligands for NET are DOTA-Phe1-Tyr3-octreotide (DOTA-TOC) and DOTA-DPhe1,Tyr3-octreotate (DOTA-TATE) widely used worldwide [36,37,38,39]. Aside from the encouraging efficacy and safety of PSMA-617, PSMA-11 and PSMA-I&T for clinical assessment of PC, efforts are ongoing towards finding unpresented radiotheranostics with better capabilities [40,41,42,43]. Gene expression profiling for prognostic and predictive issues in BC has been received considerable attention in clinic recently [7].

Choosing the patients who can benefit from radioligand therapy with these implications resulted to worldwide demand towards radiotheranostic applications for oncology management in nuclear medicine.

## 4. Targeting HER2 Receptors by Theranostics

The most striking procedure in detection and treatment was about management of BC. Since this global cancer burden affects about 49.5% of the women population and almost more than >60 years of age [4]. ERBB2 overexpression in almost all types of BC leads to the proceeding of the human epidermal growth factor receptor-2 (HER2, one subtype of HER1-4 family) [4]. It is demonstrated that the external section of the HER2 receptor has no identifiable ligand unless in dimerisation with other growth factors. The most remarkable dimer for targeting diagnostic and therapeutic purposes is the HER2-HER3 dimer [44]. Activating of these receptors motivates complicated signal transduction pathways that conduce tumorigenic proses [45]. Nowadays HER2 is a key oncogene in BC [45]. Commonly in systemic therapy approaches anti-HER2 therapy is done for HER2 positive cases [4]. Advanced methods for diagnosis and therapy of BC have been done based on HER2 as a major identified factor whose amplification leads to uncontrolled cell proliferation in breast cancer [46]. Application of anti-HER2 monoclonal antibody ‘trastuzumab’ is the most common procedure in treatment of BC [47]. Considering that this therapeutic protocol imposes patient high costs and despite that may be ineffective, assessment pre-treatment physiologic manner of the trastuzumab would be noteworthy. Also, this is demonstrated that some negative HER2 patients can benefit from therapeutic trastuzumab as an anti-HER2 agent [48]. In this regard biodistribution and accumulation of trastuzumab radiolabelled with Indium-111, [^111^In]In-trastuzumab, as an imaging agent for single photon computed emission tomography (SPECT) has been evaluated [48]. In preclinical studies, significant and specific accumulation of [^111^In]In-trastuzumab was proved in human HER2 tumour-bearing mice [49]. So, it was supposed that this tracer can be a promising agent in humans. In clinical studies of [^111^In]In-trastuzumab in women patients confirmed with HER2 positive BC and eligible for treatment with trastuzumab or paclitaxel remarkable results were obtained [50]. In 12 final cases, 25 tumour lesions were detected. Diagnostic studies were accomplished in 24, 72, 96 and 168 h after injection through scintigraphy scans. Since blood vessels are clearly visible until 72 h, for accumulation and study investigations at least 96 h interval is strongly recommended in this paper [50]. Obviously, this is evidence of a high plasma level of [^111^In]In-trastuzumab within the first 72 h of the study. Despite the competition between radiolabelled trastuzumab and therapeutic trastuzumab, the saturation effect in diagnosis is negligible. In accordance with this, it can be concluded that radiolabelled trastuzumab can be used as a diagnostic agent during the prevalent therapeutic procedures [50].

Since PET provides higher resolution and detection sensitivity so many efforts toward preparation of PET derivatives for detection of metastatic lesions of breast cancer have been devoted. Cooper-64 (^64^Cu) is a positron emitting radionuclide (β^+^, 0.653 MeV [17.8%]; β^−^, 0.579 MeV [38.4%]) and has a half-life of 12.701 ± 0.002 h [50]. These characterisations make copper-64 a qualified PET radionuclide for high-quality detection purposes. A longer half-life of copper-64 compared to other PET radionuclides makes the transportation of the final radiopharmaceutical feasible, also will be appropriate for radiolabelling of compounds with longer biological half-life such as monoclonal antibodies [50].

In an effort, [^64^Cu]Cu-trastuzumab was used clinically in 6 patients with primary or metastatic HER2 positive breast cancer [51]. All patients received intravenous (IV) injection of 130 MBq of [^64^Cu]Cu-trastuzumab and diagnostic investigations during 1, 24 and 48 h after injections were done. In this study, it was demonstrated that the sensitivity of [^64^Cu]Cu-trastuzumab in brain metastases detection can be parallel to MR imaging and even superior to CT modality in some studied cases [51]. Generally, based on this clinical trial, it can be concluded that the [^64^Cu]Cu-trastuzumab diagnostic procedure is practicable, repeatable and safe. Sensitivity of the diagnostic scan in this study was low that it can be related to trastuzumab therapy during the procedure [51].

**Figure 2:**
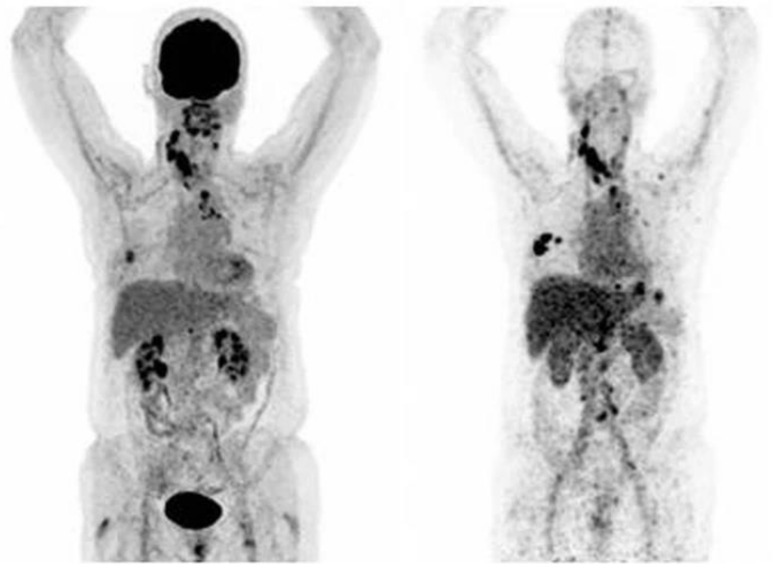
PET-Scan of [18F]FDG (left) and [^89^Zr]Zr-trastuzumab (right) of a patient with a [^89^Zr]Zr-trastuzumab scan considered HER2-positive (Figure adapted from Bensch et al. [53] Creative Commons Attribution 4.0 International License).

Immuno-PET imaging can be possible using Zirconium-89 (^89^Zr). This radiometal with a 78.4 h half-life, which is the longest half-life in the group of PET radionuclides, is completely opportune for radiolabelling of antibodies [52]. In a clinical trial performed between March 2006 and December 2008, [^89^Zr]Zr-trastuzumab (Figure 2) has been applied in 14 patients in order to assess HER2 positive lesions in metastatic BC [52]. It is supposed that 38.4 ± 1.6 MBq [^89^Zr]Zr-trastuzumab injected dose would be adequate for the evaluation of tumour uptake even 4–5 days after injection. Diagnostic procedures were done at early stages (1–3 days), also delayed imaging (4–7 days). According to this clinical trial, it is demonstrated that tumour uptake of [^89^Zr]Zr-trastuzumab is dose-dependent and tumour to non-target accumulation increases over time [52].

Significant uptakes in the liver, bone and brain lesions were reported, so based on these findings PET scan for visualisation and quantitative evaluations in HER2 positive breast cancer patients would be feasible [52]. A pilot study of [^68^Ga]Ga-DOTA-F(ab′)2-trastuzumab was investigated in 16 patients with BC [54]. In the final study 15 patients enrolled with HER2 positive (8 patients) and HER2 negative (7 patients) characterisation. Among the HER2 positive patients, all but one received anti-HER2 trastuzumab therapy. This tracer was well tolerated and the kidney was reported as a critical organ with a mean dose of 0.383 Gy/37 MBq in this study. All 15 patients had undergone [^18^F]FDG PET scan and all showed [^18^F]FDG avid abnormalities. Despite this none of seven patients with the specification of HER2 negative showed accumulation of [^68^Ga]Ga-DOTA-F(ab′)_2_-trastuzumab. In all studied HER2 positive cases (8 patients whom three of them had received trastuzumab) only seven lesions were found admirably purposeful and correlated with FDG avid lesions.

Based on these findings, it is supposed that a high concentration of trastuzumab in blood circulation may compete with the radiolabelled compound. On the other hand, it may refer to the half-life of radionuclides used for radiolabelling. As it takes time for intact antibodies for accumulating in tumour tissues, radionuclides with higher half-life are preferred. In this regard radiolabelling with indium-111, iodine-131, zirconium-89 and iodine-124 was evaluated [54]. [^124^I]I-trastuzumab and [^131^I]I-herceptin in preclinical phase studies showed optimistic results in imaging and cell culture investigations [55,56]. Also, [^99m^Tc]Tc-trastuzumab biodistribution study in tumour-bearing BALB/C female mouse whose tumour cells were established from a murine mammary carcinoma has been assayed [57]. Gamma camera imaging results showed a significant accumulation of [^99m^Tc]Tc-trastuzumab in tumours [57].

In order to develop the therapeutic counterparts of diagnostic radiolabelled trastuzumab, therapeutic radiolabelled trastuzumab derivatives are under investigation. In a pilot study feasibility of [^177^Lu]Lu-trastuzumab for therapy of HER2 positive breast cancer patients has been proven [58]. In a pilot study, 10 women patients were enrolled to investigate [^177^Lu]Lu-trastuzumab effectiveness. Each patient was injected 0.18–0.44 GBq of the radiotracer. Diagnostic evaluations were done through the administered day, also 5 and 7 days after injection. 6 HER2 positive and 4 HER2 negative patients were enrolled by [^18^F]FDG PET scan and all diagnosed with metastatic disease [58]. In a biodistribution study of [^177^Lu]Lu-trastuzumab by SPECT/CT HER2 negative cases imaging represented no tracer accumulation in the tumour sites. In contrast, imaging of HER2 positive patients was associated with significant uptake in primary and metastatic tumours. The tumour to a non-target ratio of the radiotracer uptake increased considerably during the study interval. So, the T/N was initially 2.4 on the first day of study and increased to 3.9 on the 7th day. Supported by this remarkable information it can be concluded that ^177^Lu-radiolabeled trastuzumab derivatives would be feasible in HER2 positive primary and metastatic BC patients as a palliative tracer simultaneous to other treatments [58].

Another study employing [^67^Ga]Ga-THP-trastuzumab, proved a decrease of the viability of HER2 positive specified cells in vitro. Destruction of HER2 expressive cells benefits from Auger electron irradiation by gallium-67, and based on this fact [^67^Ga]Ga-THP-trastuzumab can also be considered as a therapeutic radiotracer [59].

## 5. Targeting Gastrin-Releasing Peptide Receptor (GRPR) by Theranostic Radiopharmaceuticals

One of the considerable aspects in nuclear medicine is about the peptides, as they are related to specific receptors which are expressed/overexpressed in various type of cancers [60]. There are widespread series of peptide-based theranostic radiopharmaceuticals with common use for oncology application in nuclear medicine [13,61,62,63]. There is a lot of interest to develop radiolabelled bombesin analogues. Bombesin is a specific ligand for bombesin receptors (BnR) [64]. However, bombesin receptors include three subtypes (BB_1_, BB_2_ and BB_3_) [65], with BB_2_ (formerly known as GRPR) being the specific receptor for the gastrin-releasing peptide (GRP) and a very promising targeting vector for the diagnosis and therapy of PC and BC [60].

MacDonald et al. [66] demonstrated that bombesin is a 27 amino acid peptide and has the functional c-terminal group just as GRP. Also, GRP is known as a growth factor in normal cells as well as cancerous cells. There is evidence that BB_2_ is overexpressed in various cancer types including breast, lung, prostate, ovarian or pancreatic cancer, as well as some of the central nervous system especially glioma, meningioma and neuroblastoma [67,68,69]. Evidence has shown that in BC GRP is overexpressed in 38–96% of cases [70,71], especially in oestrogen receptor (ER)-expressing patients [72,73,74]. Based on these results bombesin moved into the focus of investigations. Until today, extensive efforts for radiolabelling of bombesin analogues with SPECT (indium-111, technetium-99m) and PET radionuclides (copper-64, gallium-68, fluorine-18) were accomplished in order to develop specific diagnostic agents for BC [60]. These radiopharmaceuticals showed optimistic results for specific accumulation in BC in preclinical studies including BC cell lines as well as animal studies [60,75,76]. Based on these promising results, many clinical trials have been done with radiolabelled bombesin receptor (BnR) agonists.

Different research groups have been conceded comparative in vivo studies using ^68^Ga- and fluorine-18 labelled BnR-agonists as PET imaging agents. These revealed higher tumour uptake in comparison to [^18^F]FDG alone in ER-positive breast cancer-bearing nude-mice [77]. Conjugation of RGD peptide for targeting of αvβ3-integrins and BnR-agonist as heterodimeric PET imaging probes showed promising potential in imaging of BC. In a preclinical study, Liu Z et al. developed the ^68^Ga-, ^64^Cu- and ^18^F-labelled RGD- and BnR-targeting radiotracers that provide great images in xenografted mice due to the high accumulation in αvβ3-integrin and BnR expressing tumour sites [78].

**Figure 3:**
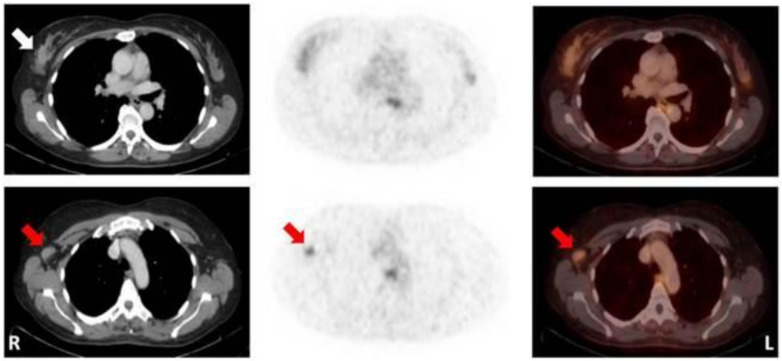
Patient with a mucinous carcinoma of the right breast with low ER/PR-expression. CT (left); ^68^Ga-RM2-PET (middle); fusion images (right); primary tumour indicated by white arrow; lymph node metastasis indicated by red arrows. (Figure unmodified from Stykow et al. [79] Creative Commons Attribution 4.0 International License).

In a limited clinical study, Stoykow et al. assessed the role of [^68^Ga]Ga-RM2 as a PET imaging tracer in 15 BC patients (Figure 3) [79]. The [^68^Ga]Ga-RM2 PET showed strongly increased uptake in 13/18 tumours compared to normal tissue. Moreover, progesterone receptor (PR) and ER expression were identified by all PET-positive primary tumours (13/13) compared to 1/5 PET-negative tumour. Which was identified as ER-positive. The others were not detected by PET. In a similar clinical study, Zang et al. discovered 29/34 primary tumours using [^68^Ga]Ga-NOTA-RM26. PET imaging proved the existence of lymph node metastases in 18 patients and positive correlations with the level of GRPR expressions were found. Moreover, the SUVmax in ER-positive tumours significantly increased in comparison to the negative tumours. It was also observed that the specificity and sensitivity of radiotracer increased in patients with ER-negative tumours, therefore these patients were excluded from this trial [80].

In another study, [^18^F]FDG was assessed in comparison with [^68^Ga]Ga-RM2 in 14 breast tumour-bearing patients by tissue micro-imaging technique. Also, Morgat et al. preformed GRPR, HER2 and Ki-67 immunohistochemistry studies for PR and ER for all cases. Results confirmed the lower [^18^F]FDG uptake in the PR-positive and specific binding of [^68^Ga]Ga-RM2 in ER-positive cancer groups. Moreover, a correlation was detected among GRPR expression in BC cells showing ER-positive tumours and indicated the diagnostic and therapeutic potential of GRPR antagonist in BC [81].

^99m^TC-based radio-biomarkers have a great potential for targeting ER/BnR. For example, ^99m^Tc-BnR agonist indicated increased uptake in breast cancer cells and enhanced accumulations in studied tumours that overexpressed receptors of GRPR in animal models [82]. Various clinical trials were conducted using ^99m^Tc-labeled BnR agonists for imaging breast lesions. Scopinaro F et al. comparatively evaluated technetium pertechnetate and ^99m^Tc-labeled Bn in three BC patients. The imaging results revealed higher uptake of ^99m^Tc-Bn tracer in the breast tumour and metastatic lymph node sites more than technetium pertechnetate [83].

Ji et al. conducted a study to investigate the use of [^99m^Tc]Tc-RGD-Bn in comparison with the ultrasound for monitoring of the tumours. Similar sensitivity and specificity for SPECT/CT and ultrasound were observed because of dual receptor targeting of the [^99m^Tc]Tc-RGD-Bn. In the case of metastatic sites the SPECT/CT was more sensitive than ultrasound. Therefore, [^99m^Tc]Tc-RGD-Bn would be considered as dual-modality imaging agent for the diagnosis of BC [84]. Similarly, [^99m^Tc]Tc-RGD-Bn, as well as [^99m^Tc]Tc-3P4-RGD2, were proven for imaging of breast lesions in six patients. Among them, five malignant lesions had distinctive uptake, and four cases have shown αvβ3-integrin and GRPR expressions [85]. In another clinical study, the physiologic distribution of [^99m^Tc]Tc-HYNIC-Lys3-Bn on 7 healthy subjects and 4 BC patients was investigated. The specific uptake was detected in the tumour site while bone marrow showed negligible uptake. Kidneys were the predominant excretion route of the radiotracer [86]. In a study, the [^99m^Tc]Tc-RP527 as a BnR-agonist biomarker was evaluated in imaging of 6 BC patients and indicated the specific tumour accumulation in 4 patients along with 6 patients [87]. Also, this research group investigated that [^99m^Tc]Tc-RP527 has a negligible uptake in tamoxifen-resistant patients.

A relatively new approach of tumour-targeting is taking into account the tumour stroma, which is representing a remarkable part of the tumoral microenvironment (TME) [88], as it affects patient prognosis and survival for several solid tumours like BC [89,90,91,92,93]. Studies could demonstrate that the tumour-stroma ratio stands in correlation with the risk to develop distant metastases and the overall patient survival [94,95]. The new focus on the TME led to further investigations that compounds of the TME may be useful targets. In this context, tumour-associated fibroblasts (CAFs) moved into the spotlight as they may be the dominating stromal cell type, depending on the cancer type [96]. Targeting CAFs can be achieved on different ways, among other things by targeting the fibroblast activation protein (FAP) which is significantly overexpressed by CAFs of numerous cancer types compared to healthy tissue [97]. The cancer-related overexpression of FAP and the significant role of CAFs in tumoral progression development of FAP inhibitors (FAPI) for cancer treatment and successively for theranostic application were logical conclusions. Recently, several preclinical as well as clinical studies with quinoline-based FAP inhibitors revealed their potential as radiotracers for tumour diagnosis [97,98,99,100,101,102]. First retrospective studies exhibited the potential of ^68^Ga-FAPI-PET/CT in comparison to ^18^F-FDG-PET/CT for a variety of tumour entities [98,103,104,105]. These results, even though further investigations are required, implicating its feasibility and advantage over ^18^F-FDG-PET/CT even though it still may not solve every of its limitations [97].

A first retrospective analysis was performed to determine the ^68^Ga-FAPI uptake in a variety of primary, metastatic or recurring cancers revealing the great potential of ^68^Ga-FAPI-PET/CT in breast cancer [106]. The highest average SUVmax was found in a group of five different cancers, amongst others breast cancer, where it outperformed ^18^F-FDG-PET/CT. Nevertheless, beside these promising results, the data on breast cancer with these new compounds are still limited [107]. Today (April 2021) there are 17 recruiting or not yet recruiting prospective trials, using FAP-specific PET, listed at clinicaltrials.gov of which at a fraction has breast cancer as condition (2/17).

**Figure 4:**
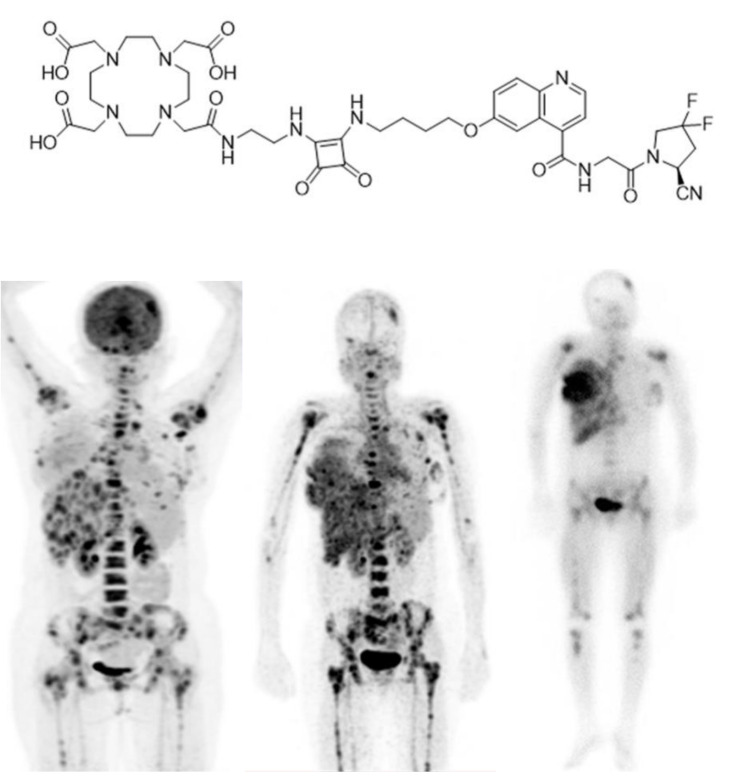
Chemical structure of DOTA.SA.FAPI. PET-Scan of [^18^F]FDG (left), [^68^Ga]Ga-DOTA.SA.FAPI (middle) and [^177^Lu]Lu- DOTA.SA.FAPI (right) of a patient with HER2-positive, (ER and PR)-negative histopathology (Figure adapted from Ballal et al. [109] Creative Commons Attribution 4.0 International License).

Recently, in another clinical trial [108], another new designed FAPI derivative named DOTA.SA.FAPI (Figure 4) was applied for diagnosis of BC in a 31 years old female patient. This new FAPI is designed as a diagnostic and therapeutic agent after radiolabelling with gallium-68, lutetium-177 (^177^Lu) or actinium-225 (^225^Ac) just like FAPI-02 and FAPI-04 [109]. Interestingly, the results indicated correlations between accumulation of [^68^Ga]Ga-DOTA.SA.FAPI and [^18^F]FDG (Figure 4) [109]. An initial patient treatment with [^177^Lu]Lu-DOTA.SA.FAPI administering 3.2 GBq was performed and post treatment imaging was accomplished 24 h after the dose injection. Conformity of dose absorption in the lesions of [^68^Ga]Ga-DOTA.SA.FAPI and [^177^Lu]Lu-DOTA.SA.FAPI is obvious and depicted in Figure 4 [109].

We tried to summarise the clinical studies listed by clinicaltrials.gov through a brief report (Table 1). A query for ‘breast cancer’ AND ‘radiopharmaceuticals’ resulted in 76 studies. For the table, only completed studies were considered while terminated, active, recruiting and not recruiting studies were excluded. Overall, 35 out of 76 listed studies were summed up. All patients included in these clinical trials were over 18 years old. Pregnant or breast-feeding patients were excluded from all trials.

Even though theranostic applications in nuclear medicine with radionuclides like gallium-68 and lutetium-177 were booming in the past decade, due to the striking development of [^68^Ga]Ga-PSMA-11 which led to the introduction of several other PSMA-targeting radiolabelled ligands for diagnosis and therapy of PC, new compounds targeting BC are rare and mostly intended for improving diagnosis. This is reflected in clinical study overview, where no therapeutic intervention can be found.

## 6. Conclusions

Undoubtedly, the use of theranostic radiopharmaceuticals has very impressive benefits in nuclear medicine which cannot be denied. Planning for targeted treatment as well as ability of simultaneous evaluation of response to treatment is a great advantage that resulted by this methodology. In the review our effort was to provide a useful summary of efforts made in the field of BC. Based on the importance of BC it is expected that more efficient theranostic agents will be developed soon. The most considerable aspect of this field is the feasibility of early detection of BC and based on that improved cancer prognosis.

## Figures and Tables

**Table 1:** An overview of completed clinical trials performed on breast cancer conducted by: clinicaltrials.gov (accessed on 20 April 2021).

Condition/Disease	Intervention/Treatment	Phase Study	Study Population (Intervention/Treatment)	Method	Actual Study Completion Date	Locations
Breast Cancer	[^89^Zr]Zr-trastuzumab	Phase 2	7 (Diagnostic)	HER2-positive lesions uptake evaluation	February 2012	University Medical Center, Groningen, The Netherlands
Breast Neoplasms, Secondary HER2 Positive Carcinoma of Breast	[^89^Zr]Zr-trastuzumab	Phase 1	20 (Diagnostic)	HER2-positive lesions uptake evaluation	September 2015	Jules Bordet Institut, Brussels, Belgium
Breast Cancer	[^89^Zr]Zr-trastuzumab	Early Phase 1	67 (Diagnostic)	HER2-positive lesions uptake evaluation	December 2017	Washington University School of Medicine @ Barnes-Jewish Hospital, Saint Louis, Missouri, United States
Breast Cancer Metastases, HER2 Positive Breast Cancer	[^89^Zr]Zr-trastuzumab	N/A	49 (Diagnostic)	HER2-positive lesions uptake evaluation	June 2020	Memorial Sloan Kettering Cancer Center, New York, New York, United States
Breast Cancer and Glioma	[^68^Ga]Ga-NOTA-NFB	N/A	46 (Diagnostic)	Evaluation of safety, biodistribution and dosimetric properties	December 2015	Xijing Hospital Nuclear Medicine Department, Xi’an, Shaanxi, China
Breast Cancer	[^68^Ga]Ga-NOTA-BBN-RGD	Phase 1	40 (Diagnostic)	Investigate the diagnostic performance and evaluation efficacy	July 2018	Peking Union Medical College Hospital, Chinese Academy of Medical Science and Peking Union Medical College, Beijing, China
Breast Cancer	[^99m^Tc]Tc-HPArk2	Early Phase 1	30 (Diagnostic)	HER2-positive lesions uptake evaluation	December 2020	Peking Union Medical College Hospital, Chinese Academy of Medical Science and Peking Union Medical College, Beijing, China
Breast Cancer	[^99m^Tc]Tc-NM-02	Early Phase 1	10 (Diagnostic)	HER2-positive lesions uptake evaluation	April 2021	Shanghai General Hospital, Shanghai, China
Breast Cancer, and others	[^68^Ga]Ga-NOTA-AE105	Phase 2	50 (Diagnostic)	Detection of lymph node metastases	March 2017	Department of Clinical Physiology, Nuclear Medicine and PET, Rigshospitalet, Copenhagen, Denmark
Breast Carcinoma	[^89^Zr]Zr-bevacizumab	Phase 2	7 (Diagnostic)	Measuring new blood vessel formation	February 2012	University Medical Center, Groningen, Groningen, The Netherlands
Breast Carcinoma	[^89^Zr]Zr-bevacizumab	N/A	2 (Diagnostic)	Measuring new blood vessel formation	March 2017	Brigham and Womens Hospital, Boston, Massachusetts, United States Dana Farber Cancer Institute, Boston, Massachusetts, United States
Breast Cancer	[^131^I]-SGMIB Anti-HER2 VHH1	Phase 1	9 (Diagnostic)	HER2-positive lesions uptake evaluation	February 2018	UZ Brussel, Brussels, Belgium
Breast Cancer	[^99m^Tc]Tc-ABH2	Early Phase 1	32 (Diagnostic)	HER2-positive lesions uptake evaluation	May 2018	Peking Union Medical College Hospital, Beijing, China
**Condition/Disease**	**Radiopahrmaceutical**	**Phase Study**	**Study Population (Intervention/Treatment)**	**Method**	**Actual Study Completion Date**	**Locations**
Metastatic Breast Cancer, and others	[^18^F]RGD-K5	Phase 2	35 (Diagnostic)	Detection and localisation of angiogenesis tissue	May 2012	University of California, Irvine, California, United States Cedars-Sinai Medical Center, Los Angeles, California, United States UMDNJ, Newark, New Jersey, United States (and 5 more...)
Breast Cancer, and others	[^64^Cu]Cu-DOTA-AE105	Early Phase 1	10 (Diagnostic)	Evaluation of uPAR (urokinase plasminogen activator receptor)	October 2014	Department of Clinical Physiology, Nuclear Medicine and PET, Rigshospitalet, Copenhagen, Denmark
Breast Tumor	[^68^Ga]Ga-NOTA-RM26	Early Phase 1	30 (Diagnostic)	Target gastrin-releasing peptide receptor in neoplastic cells evaluation	October 2018	Peking Union Medical College Hospital, Beijing, China
